# A Deep Learning Model That Combines ResNet and Transformer Architectures for Real-Time Blood Glucose Measurement Using PPG Signals

**DOI:** 10.3390/bioengineering13010049

**Published:** 2025-12-31

**Authors:** Ting-Hong Chen, Lei Wang, Qian-Xun Hong, Meng-Ting Wu

**Affiliations:** 1 Department of Electrical Engineering, Feng-Chia University, Taichung 40724, Taiwan; alan123456888@gmail.com (T.-H.C.); leiwang@fcu.edu.tw (L.W.); a0905889403@gmail.com (Q.-X.H.); 2 Division of Neurosurgery, Department of Surgery, Cheng-Hsin General Hospital, Taipei 11220, Taiwan; 3 Department of Neurological Surgery, Tri-Service General Hospital, National Defense Medical Center, Taipei 11490, Taiwan; 4 Ph.D. Program of Electrical and Communications Engineering, Feng Chia University, Taichung 40724, Taiwan

**Keywords:** photoplethysmography (PPG), non-invasive glucose monitoring, blood glucose prediction, deep learning, ResNet–Transformer hybrid model, Signal Quality Index (SQI), Clarke error grid (CEG), MIMIC-III dataset, PhysioNet

## Abstract

Recent advances in wearable devices and physiological signal monitoring technologies have motivated research into non-invasive glucose estimation for diabetes management. However, the existing studies are often limited by sample constraints, in terms of relatively small numbers of subjects, and few address personalized differences. Physiological signals vary considerably for different individuals, affecting the reliability of accuracy measurements, and training data and test data are both used from the same subjects, which makes the test result more affirmative than the truth. This study aims to compare the two scenarios mentioned above, regardless of whether the testing/training involves the same individual, in order to determine whether the proposed training method has better generalization ability. The publicly available MIMIC-III dataset, which contains 700,000 data points for 10,000 subjects, is used to create a more generalized model. The model architecture uses a ResNet CNN + Transformer block, and data quality is graded during preprocessing to select signals with less interference for training to increase data quality. This preprocessing method allows the model to extract useful features without being adversely affected by noise and anomalous data that decreases performance; therefore, the model’s training results and generalization capability are increased. This study creates a model to predict blood glucose values from 70 to 250 for 180 classes, using mean absolute relative difference (MARD) as the evaluation metric and a Clarke error grid (CEG) to determine a reasonable error tolerance. For personalized cases (specific individual data during model training), the MARD is 11.69%, and the optimal Zone A (representing no clinical risk) in the Clarke error grid is 82.7%. Non-personalized cases (test subjects not included in the model training samples) using 60,000 unseen data yields MARD = 15.16%, and the optimal Zone A in the Clarke error grid is 75.4%. Across multiple testing runs, the proportion of predictions falling within Clarke error grid zones A and B consistently approached 100%. The small performance difference suggests that the proposed method has the potential to improve subject-independent estimation; however, further validation in broader populations is required. Therefore, the primary objective of this study is to improve subject-independent, non-personalized PPG-based glucose estimation and reduce the performance gap between personalized and non-personalized measurements.

## 1. Introduction

The aging global population and lifestyle changes mean that diabetes is a significant chronic disease [1]. World Health Organization (WHO) statistics show that approximately 400 million people worldwide suffer from diabetes, and this number is expected to increase over the next decade. The International Diabetes Federation (IDF) predicts that the global number of diabetes patients will increase to 642 million by 2040 [2]. Diabetes can trigger multiple significant complications, and studies show that individuals who have diabetes and its complications demonstrate higher mortality rates after COVID-19 infection than those who do not have diabetes [3]. Diabetes significantly affects an individual’s quality of life and is closely associated with complications such as diabetic retinopathy, kidney failure, cardiovascular disease and neuropathy. Without proper blood glucose control, it can result in amputation or death. Many diabetes patients have abnormal blood glucose levels or are in a pre-diabetic state before diagnosis, but detection and intervention are often late. Early detection and prevention significantly reduce disease risk and delay progression and complications of the disease, which alleviates long-term burdens on healthcare systems and families.

Currently, the most common method for measuring blood glucose levels (BGLs) is invasive blood sampling measurement by patients or by healthcare professionals. The measurement involves pressing a lancet device against the sampling site (usually a finger) and then pressing down to collect blood. This invasive method easily causes psychological burden, pain and infection risk for patients [4]. Blood sampling typically measures single-point glucose values at a given moment, so it is not a continuous measurement method. Continuous glucose monitoring (CGM) is achieved through minimally invasive approaches using glucose sensors that are implanted subcutaneously to collect signals that are generated by chemical reactions between glucose and subcutaneous tissue fluid. This method reduces the pain of invasive blood sampling and involves smaller and shallower wounds, but it is invasive, and also expensive. Many recent studies have focused on non-invasive measurement methods.

Photoplethysmography (PPG) is a non-invasive optical technique for measuring blood volume changes [5]. In terms of the sensing method, it involves either transmission or reflection. The measurement device uses LED illumination from one end to irradiate specific patient sites, such as fingers, ears, or wrists (tissues where light easily penetrates or reflects), and a photodiode at the other end acts as the receiver. The signal contains pulsatile (AC) and non-pulsatile (DC) components [6]. The AC component is related to cardiac pulsation, and the DC component is related to the light absorption coefficient for tissues, bones, veins, and static blood. Cardiac systolic and diastolic pulse waveforms are represented using measured changes in light transmission, and PPG is widely used for measuring blood oxygen saturation and also detects cardiovascular parameters, such as heart rate (HR), blood pressure (BP) and heart rate variability (HRV) [7,8].

Recent studies have increasingly used machine-learning-based algorithms. Deep learning is used to determine the relationship between BGL and corresponding physiological signals. Compared to conventional manual computational methods to identify algorithms, machine learning allows more efficient continuous training and multi-task computational processing, which increases the accuracy with which algorithms are matched. However, PPG-based blood glucose prediction still faces several challenges. Firstly, the training/testing method used in the study on blood glucose prediction can be categorized into a personalized model and a non-personalized model. Personalized data refers to models trained and validated on data from the same group of participants, capturing individual physiological differences and enabling the model to provide a higher prediction accuracy for that specific participant. On the contrary, non-personalized data refers to models where the target participant’s data is not used for training; instead, data from other participants is used to construct the model, which is then directly applied to new participants to assess the model’s generalization ability. Due to significant differences in different individuals’ physiological signals, personalized models always provide overly optimistic and inaccurate results, precluding their practical usage. Non-personalized models are more practical in clinical applications and deployment because they do not require additional user-specific data for prediction. Secondly, while some studies have indicated that combining several physiological signals (such as PPG + ECG, blood pressure, or skin conductance) can indeed improve the accuracy of prediction, such multimodal methods often require additional sensing devices, increasing the cost and complexity, hindering convenient measurement in daily life and negating their value. Therefore, using PPG as the sole signal source remains the most practically valuable approach. Finally, because PPG signals are optical signals, they are easily affected by environmental influences such as motion, changes in lighting, and contact pressure. These artifact interferences reduce signal quality and model accuracy. To address this issue, our study focuses on data preprocessing, including noise removal, feature selection, and signal quality assessment, to avoid interfering segments and ensure that the data input into the model accurately reflects dynamic changes in blood flow, avoiding interfering segments.

In consideration of the description above, this study will focus on the following:

1. Exploring the accuracy differences between personalized and non-personalized testing, and attempting to improve the accuracy of non-personalized models;
2. Concentrating on PPG in non-invasive blood glucose measurement, rather than relying on multimodal measurements;
3. Leveraging classification of PPG signal quality in preprocessing to provide high-quality, anomaly-free PPG signals to obtain more accurate training/testing results.

Ultimately, the goal of this study is to establish a practical and accurate PPG blood glucose prediction system and verify its feasibility in non-invasive blood glucose measurement.

The structure of this paper is arranged as follows. Section 2 presents a brief survey of studies on non-invasive glucose estimation in recent years. Section 3 presents the details on the procedures and models developed in this study. The testing results are described and discussed in Section 4. Section 5 presents the conclusions of this study.

## 2. Related Works

To increase prediction accuracy, Y. Wei et al. proposed a method that uses three classical time–frequency analysis techniques [9]: Discrete Cosine Transform (DCT), Singular Spectrum Analysis (SSA) and Empirical Mode Decomposition (EMD). The results from these three methods are averaged and heart rate variability (HRV) and heart rate (HR) information are used as training features. The experimental results show that 90.59% and 86.84% of test data is within Zone A of the Clarke error grid (representing no clinical risk) for two datasets. These results are promising, but the sample size is only 26 participants, so the accuracy and applicability still require validation.

Y. Wu et al. used multi-source information fusion for glucose monitoring based on the relationship between blood glucose concentration and metabolic heat [10]. The mathematical relationship between blood glucose and metabolic heat was determined and normalization processing is used to eliminate the effect of metabolic parameters and historical measurement data. The experimental results show that prediction errors are less than ±0.25 mmol/L for a test set of 15 subjects. A similar limitation is observed in the studies mentioned above: experimental results are typically produced by a few subjects in closed experiments, which means that the actual clinical accuracy remains uncertain.

P.-L. Lee et al. developed a non-invasive glucose estimation method that uses dual-channel PPG (530 nm green light and 1550 nm infrared light) and the pulse arrival velocity (PAV) [11]. The PPG amplitude ratio at two wavelengths and PAV information are used as features for estimation. Experiments with 18 healthy subjects showed that the prediction accuracy is 100% within Zone A of the Clarke error grid. M. A. Darwich et al. proposed a non-invasive blood glucose measurement method that uses infrared light to illuminate the fingertip and read the voltage output [12]. The testing results showed that 97.5% of the sample points fell within Zone A of the Clarke error grid, 2.2% fell within Zone B, and 0.3% fell within Zone C. It should be noted that the sample size was only 30 people, including 15 diabetic patients and 15 healthy controls. Another non-invasive blood glucose monitoring method was proposed by G. Hammour and D. P. Mandic using in-ear PPG sensing [13]. PPG signals are sensed via an 880 nm infrared PPG sensor embedded in the ear to extract periodic features, and then, a machine learning model is used to estimate blood glucose levels. The study included four subjects (including non-diabetic, pre-diabetic, type 1 and type 2 diabetic patients) who were tested in fasting and postprandial situations over 9 days. The results showed that approximately 82% of the sample points fell in Zone A, while the remaining sample points were all located in Zone B. These studies all use a small sample size or analysis of individual cases, so the reliability of accuracy still requires further large-scale validation for confirmation.

S. Chen et al. used the multi-size weighted fitting (MSWF) algorithm to increase input signal reliability and avoid the boundary effects and spectral overlap that can occur during filtering [14]. Spatial position encoding and dynamic features were used to reconstruct the signal, and prior knowledge was used to determine potential physiological patterns. For a clinical dataset of 260 subjects, five-fold cross-validation shows that 87.89% of the data is within the optimal Zone A of the Clarke error grid (representing no clinical risk).

E. Susana et al. proposed a non-invasive blood glucose level classification method based on PPG signal with time-frequency analysis, which includes spectral entropy and instantaneous frequency characteristics [15]. The method collects PPG data from 219 adults in Guilin Hospital, with each segment containing 2100 sampling points. The optimal model, Super Vector Machine + Spectral Entropy, can achieve a classification accuracy of approximately 91.3%. Because the method only performs classification rather than precise value prediction, it still falls short of the current needs for continuous blood glucose monitoring and clinical applications.

V. B. Srinivasan and F. Foroozan et al. used the large public database MIMIC-III [16,17,18] and convolutional neural network (CNN) classification models to categorize subjects into diabetic and non-diabetic groups and used the model to predict whether individuals have diabetes [19]. The training phase uses 584 data points, and the validation data uses personalized data for verification. The experiments show that using only PPG signals for training achieves a model accuracy of 72.76%, but if other physiological signals (blood pressure and age) are used as additional features, the model accuracy increases to 76.34%. Therefore, adding extra physiological features to the original PPG signals increases the model performance. This study is commendable because its training and validation data are drawn from a publicly available data set, and the amount of data is also relatively larger than that of others. This makes its experimental results more reliable than those of other related studies. However, the study makes the same mistake as most other related studies—its validation data is derived from individuals with the same training data, that is, using personalized data for validation. This produces an artificially high accuracy and increases the risk of diagnosis.

It should be noted that F. Gómez-Peralta evaluated a commercially available non-invasive blood glucose monitoring system, Glucube^®^ [20], which is based on near-infrared spectroscopy. A total of 105 subjects (including those with diabetes, prediabetes, and normoglycemia) were included in the evaluation, and the data measured by the device were compared with the fingertip blood glucose values. The results showed that the overall MARD was 20.3%, of which the MARD for the blood glucose value above 100 mg/dL was 18.3%, and more than 95% of the data fell into the A + B zone. This survey showed that the blood glucose detection accuracy of similar products on the market has not yet reached the ideal level, and their performance is somewhat inferior to that of the abovementioned study.

## 3. Research Methods and Procedures

In reviewing the literature, the following three key issues are identified:

**(1) Insufficient training data volume:** Most studies use self-developed devices to collect blood glucose samples, so training samples are small. Prediction accuracy seems greater when results are viewed individually, but generalizability is poor, so its performance for non-personalized samples is worse than has been claimed.

**(2) Lack of data preprocessing:** Previous studies do not rigorously analyze or process acquired data, so training models are misled by noise interference, and prediction accuracy is poor. In particular, in the acquisition of PPG signals, light is highly sensitive to the external environment. Even a slight vibration or touch immediately produces motion artifacts and causes data to be distorted.

**(3) Lack of analysis of differences between personalized and non-personalized data:** Most studies use data for specific individuals during model training for model validation, but this is personalized data. Individual differences produce different variations in PPG signals for different individuals, so data for individuals whose information is not used for model training (non-personalized data) is used for validation to objectively assess the model’s ability to be generalized.

### 3.1. Data Collection

This study shows that data has a significant effect on research outcomes. Using self-designed devices to collect data imposes significant research limitations if large amounts of training data are unavailable; therefore, models do not achieve generalizable results during training, and deep learning is difficult to implement for non-invasive blood glucose measurement. This study uses the publicly available MIMIC-III Waveform Database Matched Subset dataset from the United States, and uses 6238 test subjects as the training set data and 2337 subjects as the test data (personalized data). This database contains approximately 10,000 ICU (intensive care unit) patients, and each file includes .dat and .hea files, for waveform data and matching data content annotations, respectively. All samples have a sampling frequency of 125 Hz. This study uses 10-s lengths as one unit to create one-dimensional arrays with 1250 sampling points.

Metavision (Metavision, iMDsoft Ltd., Wakefield, MA, USA) as the bedside monitoring system for data collection to allow for more accurate data collection. MIMIC-III uses more complete physiological features than MIMIC-II and uses Metavision as the bedside monitoring system for data collection to allow for more accurate data collection. In terms of blood glucose values, blood glucose data requires controlled access; therefore, PhysioNet’s Curated Data for Describing Blood Glucose Management in the Intensive Care Unit [21,22] dataset is used. Paired PPG signals from patients with the same patient IDs as those in the MIMIC-III Waveform Database Matched Subset are extracted, and blood glucose values for these PPG signals are labeled.

### 3.2. Preprocessing

To construct PPG segments corresponding to sparse blood glucose measurements, a window-based temporal extraction strategy was applied prior to signal preprocessing. Because blood glucose values are infrequently measured in clinical practice, with most patients having only two to three measurements per day, assigning a single short PPG segment to each measurement would provide a limited physiological context and insufficient training samples. Therefore, a ±9-min interval surrounding each blood glucose measurement was treated as a physiological state window representing the local cardiovascular condition associated with that measurement. This window was subsequently segmented into multiple 10-s PPG intervals for model training.

This design does not assume that each PPG segment within the window precisely corresponds to the measured glucose value. Instead, the window captures the surrounding physiological context, allowing the model to learn meaningful temporal patterns from continuous PPG signals under sparse glucose supervision. Temporal alignment was performed strictly according to the blood glucose timestamps provided by the MIMIC dataset and the PhysioNet curated glucose records. For each measurement, PPG signals from the same patient were extracted from 9 min before to 9 min after the measurement time whenever available; otherwise, the longest continuous segment was used. All alignment procedures followed the official records to ensure reproducibility.

PPG signal preprocessing has three stages: missing value processing, filtering processing and SQI grading. When graded data (A~F) is created, data analysis and organization are performed to complete preprocessing.

#### 3.2.1. Missing Value Processing

**Figure 1:**
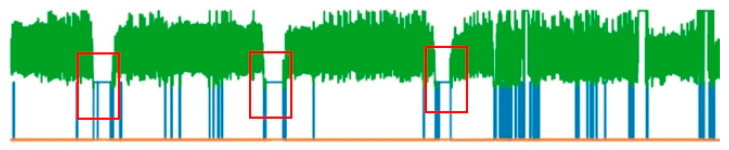
Missing value illustration.

In Figure 1, the green line represents the PPG signal, the blue line segments below indicate where PPG-signal missing values exist and the red boxes mark areas of long-term missing data. During missing value processing, these long-term missing segments are deleted.

There are two categories of missing values: the first likely occurs for the same reason as flat lines and flat peaks—exceeding the upper limit of the instrument’s measurement range. This type of signal is maintained to preserve the waveform. The second category, as shown in the red boxes in Figure 1, involves long-term data loss, which is removed. To preserve complete waveforms, missing value signals are filled and interpolated. To maintain physiological meaning without over-interpolation, heart rate frequency is used to set thresholds. Normal heart rate is converted to sampling points with approximately 53~187 sampling points per heartbeat and waveform peaks or troughs account for about 1/4 of the entire wave, which is 13~47 sampling points. The missing value threshold is established as 30 sampling points, and segments within the threshold are filled and interpolated to preserve the original waveform.

#### 3.2.2. Filtering Processing

This study extracts pulse data and filters interference and noise using filtering processing for the original PPG signals. The Butterworth bypass filter is used because it has a flat passband response and moderate transition band characteristics, so it preserves the important frequency components in signals. The frequency range is 0.5 Hz to 10 Hz to extract main pulse signals and suppress respiratory signals below 0.5 Hz and electromagnetic interference noise above 10 Hz. During the filtering process, PPG signals first undergo bandpass filtering to remove unwanted low- and high-frequency noise, and only effective signals within the target frequency band remain. Z-score normalization of the filtered signals standardizes signals to a zero mean and unit standard deviation. This reduces inter-sample variability, thereby enhancing the signal quality and analytical reliability.

#### 3.2.3. SQI Grading

PPG signals are optical signals, so even slight noise or interference from respiratory signals, motion-induced interference (i.e., motion artifacts) and poor measurements due to lost instrumentation affect results. These noises can affect blood glucose prediction results, so directly collecting cardiovascular features from data with interference and noise can cause bias. Before conducting PPG signal analysis, signal quality must be determined to ensure proper handling of signals that are corrupted by noise interference and artifacts.

After missing value processing and filtering processing, PPG signals exhibit varying degrees of distortion, so signal quality is classified using the Signal Quality Index (SQI) that was proposed by Shao Hanyu et al. [23] for statistical analysis of PPG signals. This index is used to detect motion artifacts and to classify signal quality. Signals for different individuals vary due to individual differences, but waveforms for individuals that are measured over short time periods do not change significantly, so statistical analysis of PPG signals is used to detect and assess motion artifacts. The method marks peaks and troughs, segments each wave and calculates skewness (1), kurtosis (2) and standard deviation (3) for each wave as follows:

(1) $$skewness=\frac{\frac{1}{n}\sum_{i=1}^{n}{\left(x_{i}-\bar{x}\right)}^{3}}{{std}^{3}}$$

(2) $$kurtosis=\frac{\frac{1}{n}\sum_{i=1}^{n}{\left(x_{i}-\bar{x}\right)}^{4}}{{std}^{4}}$$

(3) $$std\left(standard\right)=\sqrt{\frac{1}{n}\sum_{i=1}^{n}{\left(x_{i}-\bar{x}\right)}^{2}}$$

After calculating the skewness, kurtosis and standard deviation for each wave, the average values for these three indicators are established as thresholds $T_{k}$, $T_{s}\;$ and $T_{\sigma }$:

(4) $$T_{k}=\bar{k}+a_{1}$$

(5) $$T_{s}=\bar{s}+a_{2}$$

(6) $$T_{\sigma }=\bar{\sigma }+a_{3}$$

**Figure 2:**
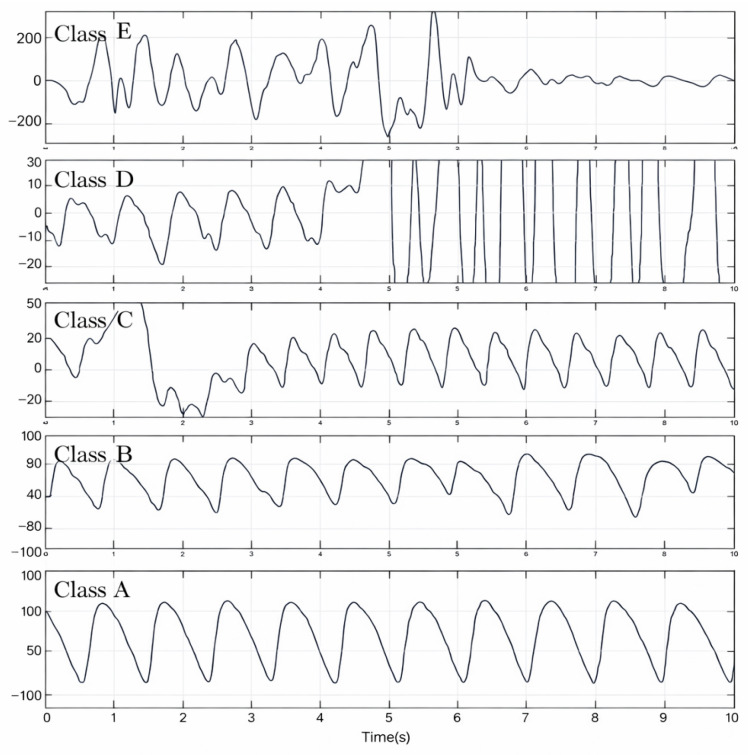
Signal quality classification.

Based on the experimental results, the value of $a_{1}$ is established as 3.1, $a_{2}$ values are 0.8 and −0.3, and $a_{3}$ is 3.2. Data is segmented every 10 s; quality statistics are collected for waves covered within 10 s, and each wave is classified into six grades from A to F. If all wave indicators within ten seconds are within the thresholds, the result is Grade A. If 0–20% of wave indicators within ten seconds are outside the thresholds, the result is Grade B. High-quality PPG signals exhibit an approximately uniform pulse morphology. Figure 2 shows that signals that are lower than Grade C contain significant noise components that are difficult to repair.

#### 3.2.4. Methods and Improvements

**Figure 3:**
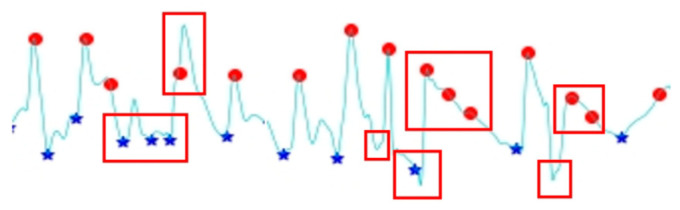
Problems with the peak and valley detection method of a previous study.

In our previous study on improving signal preprocessing to improve the accuracy of blood pressure prediction [24], we found three areas for improvement. In terms of the moving step size, the step size must be limited to prevent excessive jumps that skip the next peak or valley, or to prevent steps that are too small, which do not identify correct point movement. The search window also may not contain actual peaks and valleys. If the window does not cover the points where peaks and valleys exist, the maximum or minimum values in the window are incorrect. Peaks and valleys may also be identified incorrectly due to the presence of dicrotic waves in PPG waveforms. This method easily results in the misidentification of peaks and valleys. These three problems are illustrated in Figure 3.

Each of these three problems is addressed separately. Peaks in heart rate frequency and PPG waveform characteristics are determined by changing the moving step size to 90 points if it is less than 90 points and to 150 points if it exceeds 150 points. To search for valleys, the moving step size is forced to 60 points if it is less than 60 points, and to 150 points if it exceeds 150 points. This ensures that all moving starting points are within physiologically reasonable ranges without excessive or insufficient movement.

In terms of the search window, the values before and after a peak must be smaller, and vice versa. A discriminant is used to determine whether there are points before and after the identified peaks and valleys that are more suitable as peaks or valleys.

In terms of incorrectly identifying peaks and valleys, PPG waveform characteristics are used, and the identified target peaks and valleys are run through a correction algorithm for adjustment. The algorithm requires that normal continuous waveforms exhibit alternating peaks and valleys. If two consecutive peaks appear without an intervening valley, it is necessary to determine whether a valley was missed due to detection error. If no valley is missed, the higher peak is retained. If two consecutive valleys have a missing peak between them, a similar inspection for missed detection is required. If there is none, the left valley may be a dicrotic wave valley, so the left valley is deleted, and the right one is retained. Other waveforms are processed using the same principle.

The results show that many clearly visible interferences are not detected, so problematic signals are classified as clean signals and grading is poor. The threshold constants are often too wide. PPG signals have non-fixed values, so threshold constants must change as the magnitude of values changes. Appropriate thresholds must be assigned to address this problem. The standard deviation threshold is modified according to PPG characteristics as

(7) $$T_{\sigma }=\bar{\sigma }*\;a_{3}$$

This adjustment is necessary because, unlike skewness and kurtosis, standard deviation values vary significantly with data differences. Thresholds must be adjusted according to their own magnitude, rather than having fixed values. The experimental results show that optimal performance is achieved if $a_{1}$ is 1.5, $a_{2}$ values are 0.6 and −0.4 and $a_{3}$ is 1.

Outlier thresholds were assigned based on the PPG values after z-score normalization, which are greater than 4 or less than −4, and values with an amplitude difference exceeding 8. After improvements, the number of thresholds increases from 4 to 9, and these thresholds are used for secondary data grading to achieve more precise SQI data classification. Experimental results confirm that the improved preprocessing method can achieve more accurate SQI data classification and obtain higher accuracy.

### 3.3. CNN Model Architecture and Training Strategy

The proposed model consists of three major components: a ResNet50-based CNN encoder for local feature extraction, two Transformer blocks for modeling long-range temporal dependencies, and an attention-based fusion layer that integrates additional physiological inputs. An overview of the architecture is shown in Table 1.

The model is trained using 10-s PPG segments (1250 samples per window) paired with corresponding glucose values. Additional physiological inputs (previous glucose values, rate of change, glucose level classification) are concatenated in the attention fusion stage. The training set contains 6238 subjects, while personalized validation uses separate windows from the same subjects, and the non-personalized test set consists of 2337 unseen subjects. This summarizes the dataset configuration relevant to the model architecture and training process.

**Figure 4:**
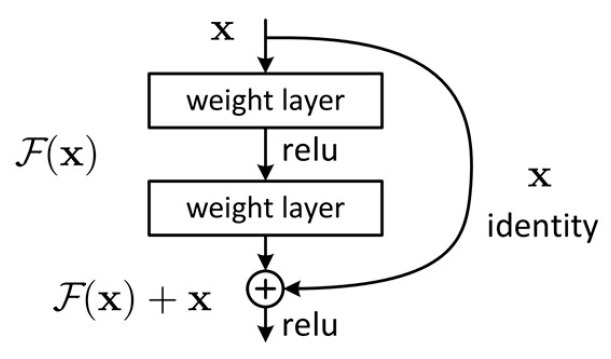
Skip connections perform element-wise addition using convolutional layers, not direct connection.

Because PPG signals exhibit distinct periodicity and local shape characteristics on the time axis, their structure resembles the edge or texture features in images. These waveforms contain different physiological meanings at different time scales and therefore can be considered as a one-dimensional local time–frequency structure. For extracting the characters, this study uses a convolutional neural network (CNN) with a ResNet50 (Residual Network 50) structure as the core model for extracting and analyzing features in PPG signals. Using VGG, ResNet50 adds skip connections that reduce gradient vanishing without adding any parameters. Skip connections perform element-wise addition using convolutional layers, rather than a direct connection, as shown in Figure 4. The model learns the residual between input and output, which significantly reduces the number of model learning parameters and the difficulty. Residual blocks use a bottleneck architecture, decomposing one 3 × 3 convolution kernel into a group of (1 × 1, 3 × 3, 1 × 1) convolution kernels, which reduces the use of model parameters. ResNet ensures that deep neural networks can be trained and prevents the use of an excessive number of parameters, even for deep models.

Convolutional neural networks are inherently local operations that capture discriminative local features in PPG signals using fixed-size convolutional kernels. PPG signals are temporal sequences, so analyzing only local features can cause temporal characteristics and information to be overlooked, and global information and contextual dependencies are not captured in the sequence.

Recent biomedical signal processing research has shown that attention-based architectures are particularly effective when the target task requires integrating information across multiple spatial or temporal scales. Hayat M. et al. demonstrated that attention mechanisms can selectively weight salient feature regions, enhance cross-region interactions and refine feature boundaries in complex medical data [25]. Such findings indicate that attention-based models are well suited for sequential physiological signals like PPG, where long-range temporal relationships and subtle waveform variations must be jointly modeled.

This study uses Transformer blocks after ResNet50, as shown in Table 1, to enhance the model’s ability to learn long-range temporal dependencies. Using self-attention mechanisms, the model dynamically captures correlations between arbitrary positions in the sequence without the limitation of a fixed receptive field. Transformer blocks compensate for ResNet50’s shortcomings in terms of temporal sequence learning, so the model uses local and temporal features from all time points. This hybrid architecture extracts local features from PPG signals using ResNet50 to produce stable and discriminative primary features, and Transformer blocks use full-sequence features to capture long-term trends and contextual implications. This hybrid architecture maintains the advantages of convolutional networks in terms of local feature extraction and uses the sequential learning capability of Transformer blocks to increase the model’s overall performance and generalization ability.

This model uses ResNet architecture and Transformer blocks in a total of 16 convolutional layers, 3 pooling layers, residual modules and self-attention mechanisms.

The model initially processes the input using a 1D convolutional layer with 64 kernels of size 7 × 1, followed by a 3 × 1 max pooling layer to reduce the temporal dimension. Information then enters the ResNet main structure, which consists of multiple residual modules to extract multi-branch features. There are four stages that progressively increase the number of channels (256, 512, 1024, 2048). The first module of each stage performs down-sampling to reduce the number of hierarchical features.

**Figure 5:**
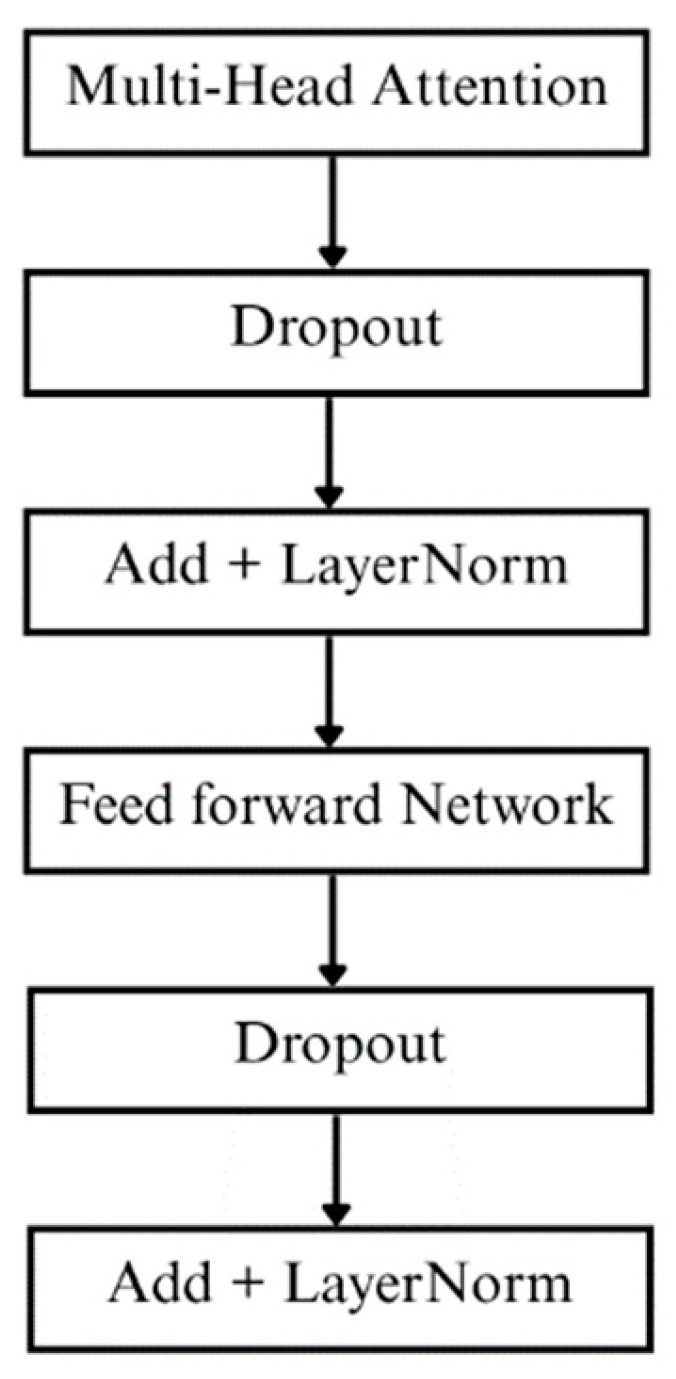
Transformer block structure.

After ResNet feature extraction, the output proceeds to two cascaded Transformer blocks, as shown in Figure 5. Using Multi-Head Attention mechanisms and residual connection structures, these blocks capture long-range temporal dependencies in the sequence to compensate for the shortcomings of convolution during temporal modeling.

The model then uses a global average pooling mechanism to compressing the entire sequence of features into a single vector, fusing with additional six-dimensional features (including previous blood glucose values, the time interval, grading information, and the rate of change), which then passes through fully connected and softmax layers to complete the 180-class classification task for glucose ranges from 70 to 250 mg/dL. The results demonstrate that using these additional features and modifying the model results in a significant decrease in the MARD value for non-personalized data, so the model’s generalization capability is increased.

To enhance model accuracy and the generalization capability of glucose measurements, several optimization strategies are used during the training phase, including label mechanisms that are paired with loss functions, data processing and adjusting the learning rate, all of which are tailored to actual data characteristics and task requirements. In terms of the design of the label mechanism and the loss function, this study uses a soft label distribution strategy and not traditional one-hot encoding. For each sample’s corresponding true glucose value, probability weights are assigned to neighboring tolerance interval categories, so the model learns a smooth probability distribution, rather than a single correct answer. Specifically, the center category receives the maximum weight (0.5) and the weights for adjacent intervals decrease linearly by distance. This weight is paired with the Kullback–Leibler divergence (KL divergence) as the loss function to enhance the model’s tolerance to prediction errors. This enhances the stability of boundary samples. In terms of the model training strategy, to avoid a premature decrease in the learning rate, which creates local minima, a Delayed ReduceLROnPlateau mechanism is used, learning rate adjustments are suspended during initial epochs and learning rates are reduced only after validation loss stabilizes. This increases convergence efficiency and model stability. An early stopping mechanism that automatically terminates training when validation loss fails to increase over several consecutive training epochs is used to prevent overfitting and conserve training resources. These training strategies correspond to data characteristics and tolerance ranges to allow the model to learn trends in glucose variation more stably. Model development and training were implemented using Python with TensorFlow version 2.10.0 (TensorFlow, Google LLC) and NumPy version 1.23.5 (NumPy Developers). All models were trained using an NVIDIA GeForce RTX 4070 GPU (NVIDIA Corporation, Santa Clara, CA, USA).

## 4. Evaluation and Results

### 4.1. Validation Methods

The validation methods primarily use Clarke error grid analysis and MARD (mean absolute relative difference) for the validation of experimental results.

**Figure 6:**
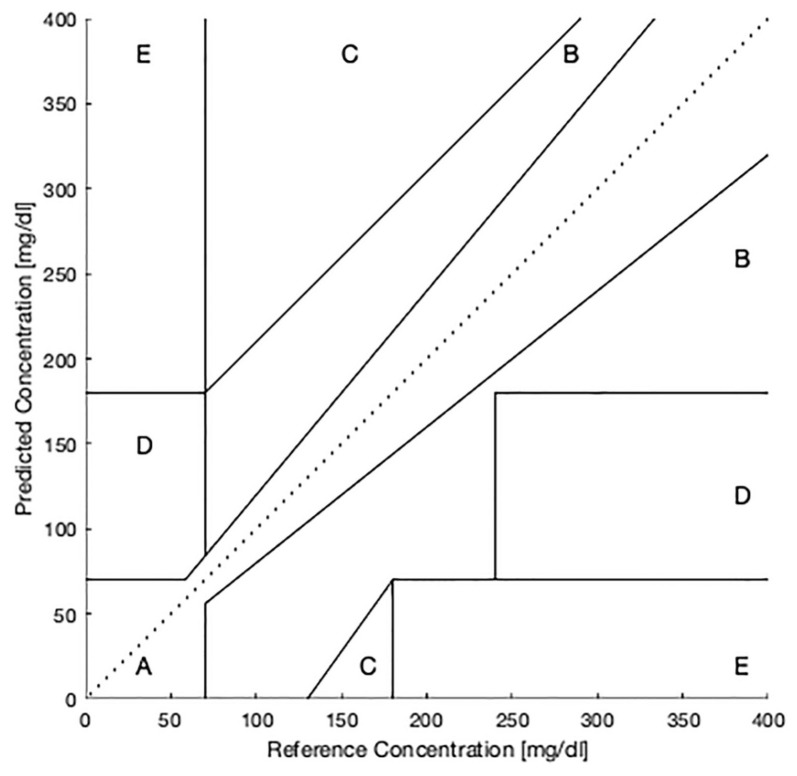
Clarke error grid analysis.

Clarke error grid analysis (EGA) [26], as shown in Figure 6, was developed in 1987 to confirm the accuracy of blood glucose estimations from glucose meters or other devices. The *Y*-axis represents the measured blood glucose value and the *X*-axis represents the actual blood glucose value. In the grid, Zone A represents the measurement and actual value error within 0–20%, Zone B represents 20–40%, Zone C represents 40–60%, Zone D represents 60–80% and Zone E represents 80–100%. This method uses five levels, but clinically acceptable error ranges are in Zone A or Zone B, and other zones are not suitable. This method was a gold standard for determining the accuracy of glucose meters or glucose systems in 1987.

MARD is the mean absolute relative difference. This is an indicator to evaluate the accuracy of continuous glucose monitoring systems. It is calculated as follows:

(8) $$MARD=avg\left(\frac{\left|Measured\;value-Reference\;value\right|}{Reference\;value}\right)\times 100\%$$

The blood glucose values that are measured using instruments are compared with reference blood glucose values (usually laboratory-measured blood glucose values). The absolute value of the difference between the two is calculated as a proportion of the reference value, and the average for all samples is calculated. This is multiplied by 100% to be expressed as a percentage. MARD is currently the most commonly used indicator for evaluating the accuracy of continuous glucose monitoring systems, and measures the consistency and stability of the overall measurement system across the entire blood glucose range, especially for extremely high or low blood glucose intervals, for which it demonstrates risks of model inaccuracy and provides a quantitative value relative to clinical standards.

For previous studies and industrial products, MARD values of less than 10% typically apply to systems that are highly accurate and are suitable for use in clinical decisions; values between 10 and 15% represent moderate accuracy with preliminary application potential. MARD is the core indicator for evaluating model prediction performance and an important quantitative basis for the development, validation and clinical application of continuous glucose monitoring systems and for measurement of the accuracy of CGM systems.

In the early-stage experiments using the pure ResNet50 model, we randomly selected 4157 subjects from the MIMIC dataset as training data, resulting in a total of 200,000 5-s PPG segments. Among these samples, 80% were used as the training set and the remaining 20% as the personalized validation set. The non-personalized test set consisted of 1846 subjects independent of the training set, totaling 50,000 5-s PPG segments. During this preliminary phase, the ResNet50 model was trained using 5-s PPG inputs (sampling frequency of 125 Hz, 625 samples per segment) to predict blood glucose values between 70 and 250 mg/dL. However, due to the significant individual differences within non-personalized data and the requirement for continuous measurement, the non-personalized results in these early experiments primarily relied on MARD as the accuracy criterion.

### 4.2. Results

Before presenting the experimental results, it is important to highlight the primary performance endpoint of this study. A key metric of interest is the non-personalized MARD, which reflects the feasibility of subject-independent glucose estimation—a central objective of this work.

In addition, before discussing the final results, it is necessary to clarify the data input to the model. In our preliminary study (before incorporating the Transformer), we investigated the impact of SQI grading on model prediction performance. To determine the effectiveness of SQI grading, the following four groups of data are used for 30 epochs of training:

(1) Pure Grade A data (170,000 data points);
(2) Grade A + B data (120,000 Grade A data points + 50,000 Grade B data points);
(3) Grade A + B + C data (95,000 Grade A data points + 50,000 Grade B data points + 25,000 Grade C data points);
(4) Ungraded data (170,000 data points).

Table 2 shows the MARD obtained on the training set/personalized data/non-personalized data when training with different quality levels of data. It can be observed that, although SQI grading has a minimal impact on non-personalized data, it reduces the MARD by approximately 10% for personalized data (a lower MARD indicates better performance).

**Figure 7:**
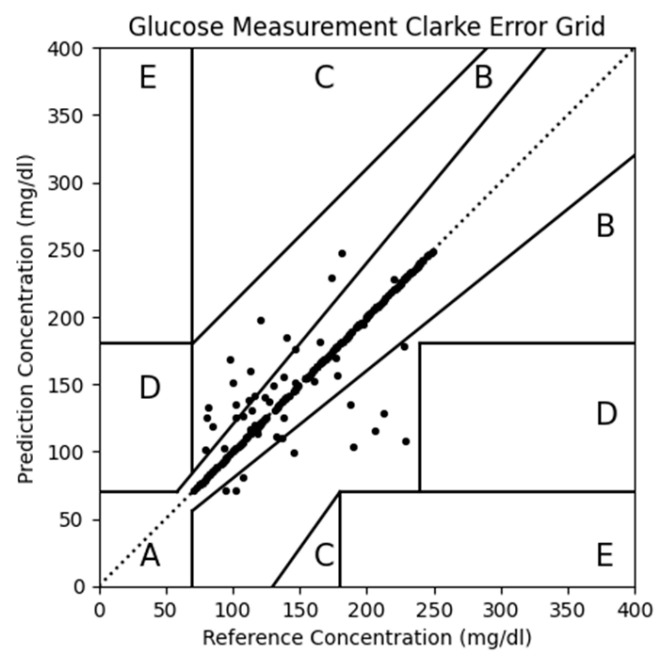
Personalized Clarke error grid.

We also conducted Clarke error grid analysis, as shown in Table 3 and Figure 7. It can be observed that pure Grade A data achieved the highest proportion in Zone A, with the combined proportion of Zone A and Zone B reaching 99.2%. Whether in terms of MARD or Clarke error grid analysis, pure Grade A data demonstrated superior performance. Therefore, in subsequent experiments, we exclusively used Grade A data.

During the research process, we discovered that the model was overly biased toward personalized data. We hypothesized that this might be related to the sample size and the blood glucose distribution range of the personalized data. Therefore, we made adjustments to the data in these two aspects. First, due to the model’s excessive bias towards personalized data, we decided to increase the training sample size from the original 4157 subjects to 9018 subjects, totaling 370,000 PPG signals. Additionally, to accommodate subsequent model modifications, we extended the training and validation data from the original 5 s to 10 s to capture more temporal features. Second, to address the distribution issue, after completing SQI grading, we first split 20% of the samples from each blood glucose value in the training data as validation data. We then analyzed the distribution of all training samples and found that the sample distribution within our prediction range of 70–250 followed a normal distribution. To enable the model to learn more comprehensively during training, reduce result bias caused by the training sample distribution and enhance the model’s sensitivity to regions with fewer samples, we used the average number of samples across all blood glucose values as a baseline. For ranges where the sample count exceeded the overall average, we randomly discarded excess samples, retaining only the average quantity, thereby reducing the impact of personalized data distribution on the model’s generalization capability.

Table 4 shows the MARD values on training, personalized, and non-personalized data for pure ResNet, the model with Transformer integration and other improvements. It can be observed that although the MARD for personalized data increased slightly, the MARD for non-personalized data decreased by 4%, indicating that we successfully improved the model’s generalization capability.

**Figure 8:**
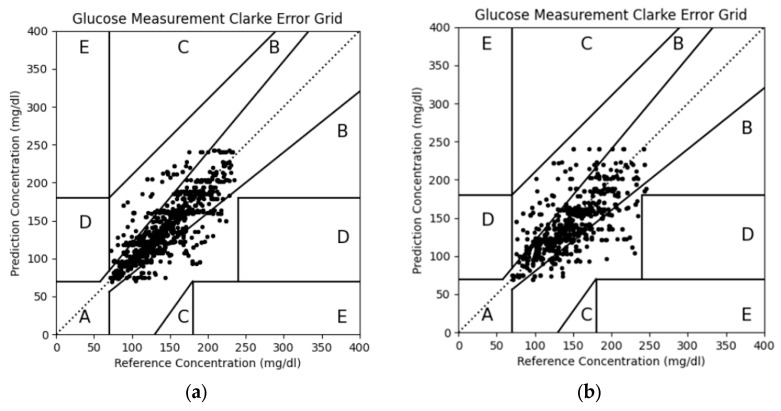
(**a**) Personalized Clarke error grid; (**b**) non-personalized Clarke error grid.

The Clarke error grid analysis also demonstrated that our improvements achieved good generalization capability, as shown in Table 5 and Figure 8. The measurement results for both personalized and non-personalized data all fell 100% within Zone A and Zone B, meeting the clinically acceptable range. This demonstrates the feasibility of our proposed method in terms of accuracy and safety.

## 5. Conclusions

This study constructs a deep learning model that uses PPG (photoplethysmography) signals and minimal historical glucose information to allow real-time, non-invasive glucose measurement to increase user convenience and ease of health monitoring. A hybrid neural network architecture uses ResNet50 and Transformer blocks. The front-end uses ResNet50 for local feature extraction and multi-scale construction, the middle section uses two Transformer blocks to model global dependencies in time series using multi-head self-attention mechanisms, and the back-end uses attention pooling with multi-dimensional additional physiological information (including previous and second-previous glucose values, time intervals, rate of change and glucose levels) to enhance the model’s ability to determine the current glucose status.

In terms of data processing, this study categorizes the original PPG signals and uses a Signal Quality Index (SQI) classification mechanism to increase data accuracy. Experiments demonstrate that high-quality Grade A signals substantially improve personalized measurement accuracy by enabling more effective feature learning. A soft label design is used for tolerance interval target distributions to give the model greater flexibility and stability for boundary predictions. To address data imbalance issues, median filtering and balance processing are used to ensure that the model maintains good performance for clinically critical ranges, such as hypoglycemia and hyperglycemia.

To increase the model’s generalization capability, this study increases the measurement accuracy for non-personalized data. Additional information, including historical glucose values, time intervals, rate of change and glucose classification, is used to allow the model to better analyze the characteristic patterns of PPG signals for different states, so personalized and non-personalized data give more similarly accurate results. A model that uses Transformer blocks demonstrates more stable generalization capability than traditional CNN architectures for validation and test sets and is particularly suited to time-related trend changes. This shows the importance of temporal attention modules for glucose sequence modeling. Data quality management, structural innovation, data augmentation and the use of multi-dimensional input information significantly contribute to model performance.

In conclusion, this study successfully establishes a glucose measurement model that uses convolutional and attention mechanisms and verifies its feasibility for non-invasive PPG signal analysis and real-time glucose measurement. The hybrid network architecture uses ResNet50 and Transformer blocks to balance local and global feature extraction. Using multi-dimensional additional physiological information and data quality classification strategies significantly increases measurement accuracy and model stability. The main contributions are as follows:

**(1) Diverse Sample Sources:** The training and validation data for this study encompass a large number of individual samples, so the study does not rely on only a few cases for modeling. This increases the model’s generalization capability and the credibility of research results.

**(2) Reducing the Performance Difference between Personalized and Non-personalized Measurements:** Decreasing the performance difference for personalized and non-personalized models increases the clinical feasibility and practicality of glucose monitoring systems. Previous studies show that non-personalized models are significantly less accurate for unobserved individuals and do not account for physiological differences and glucose fluctuations between different users. This study uses a feature input strategy that uses multiple contextual information in terms of historical glucose values (such as the previous two glucose values), time intervals, glucose change rates and glucose level classifications to increase the model’s sensitivity to glucose change trends and individual differences.

Experimental results show that this feature enhancement strategy leads to modest improvements in measurement performance for non-personalized models. The MARD value, which is a key indicator for measuring continuous glucose monitoring accuracy, decreases from 19.17% to 15.16%. This significantly decreases the difference in accuracy for personalized and non-personalized models and demonstrates that using features that represent temporal and dynamic change information increases the generalization capability. This study’s method represents a foundation for the practical deployment of glucose measurement models, reduces the dependence on large amounts of individualized data and increases the model’s real-time applicability and reliability for different users.

**(3) Model Architecture and Improved Time Series Modeling:** This study represents one of the early applications of a ResNet50 and Transformer block fusion architecture for PPG-based glucose prediction. By incorporating time-dependent features, the proposed approach achieves near-complete placement within Zones A and B of the Clarke error grid across repeated evaluations for both personalized and non-personalized data, indicating clinically acceptable accuracy and safety.

This study establishes a glucose measurement model that uses convolutional and attention mechanisms and verifies its feasibility for non-invasive PPG signal analysis and real-time glucose measurement. This study proposes technical innovations and provides preliminary evidence supporting the feasibility of non-invasive, PPG-based glucose estimation. Further validation in outpatients, wearable devices, and prospective clinical settings will be required before broader clinical application should be considered. Although the proposed method reduces the non-personalized MARD to approximately 15%, this accuracy level remains higher than what is typically achieved by commercially available CGM systems, which often report MARD values below 10%. Therefore, a non-personalized MARD of this magnitude is more suitable for trend monitoring or screening applications rather than direct clinical decision-making. Nevertheless, the ability to achieve stable performance across individuals represents an important step toward practical deployment, and further reducing non-personalized MARD toward the accuracy observed in commercial CGM devices remains a key direction for future studies. Overall, the clear improvements in subject-independent generalization highlight the potential broader real-world applicability of the proposed framework.

## Figures and Tables

**Table 1:** Architecture for ResNet50 with cascaded Transformer block model.

Layer Name	Layer Size
Input	(1250, 1)
Conv1	[7 × 1, 64, s = 2]
Max pool	[3 × 1, s = 2]
Conv2_x	$\left[\begin{array}{cc} 1\;\times \;1 & 64\\ 3\;\times \;1 & 64\\ 1\;\times \;1 & 256 \end{array}\right]\;\times \;3$
Conv3_x	$\left[\begin{array}{cc} 1\;\times \;1 & 128\\ 3\;\times \;1 & 128\\ 1\;\times \;1 & 512 \end{array}\right]\;\times \;4$
Conv4_x	$\left[\begin{array}{cc} 1\;\times \;1 & 256\\ 3\;\times \;1 & 256\\ 1\;\times \;1 & 1024 \end{array}\right]\;\times \;6$
Conv5_x	$\left[\begin{array}{cc} 1\;\times \;1 & 512\\ 3\;\times \;1 & 512\\ 1\;\times \;1 & 2048 \end{array}\right]\;\times \;3$
Transformer block 1	Multi-Head Attention (4 heads), FFN: (256→2048)
Transformer block 2	Multi-Head Attention (4 heads), FFN: (256→2048)
Global avg pool	[1 × 2048]
Info input	(6)
Dense (info)	(6→256)
Concatenate	(2048 + 256)
Dense (fusion)	(1256)
Fully connected layer	(256→180, softmax)

**Table 2:** Effect of SQI grading on MARD (%) using three datasets: training, personalized (same individuals used in training), and non-personalized (individuals not seen during training).

	Ungraded	Grade A	Grade A + B	Grade A + B + C
Training	7.23%	5.52%	5.13%	7.17%
Personalized	19.11%	9.25%	11.23%	13.72%
Non-personalized	19.26%	19.17%	22.49%	23.14%

**Table 3:** Clarke error grid analysis (personalized).

	Zone A	Zone B	Zone C	Zone D	Zone E
Grade A	91.01%	8.19%	0.58%	0.22%	0%
Grade A + B	87.44%	11.61%	0.75%	0.14%	0.06%
Grade A + B + C	82.39%	16.28%	0.96%	0.37%	0%
Ungraded	78.13%	20.34%	0.85%	0.67%	0.01%

**Table 4:** MARD (%) results before and after model enhancement, evaluated on three datasets: training, personalized and non-personalized.

	ResNet	ResNet + Transformer Block
Training	5.52%	11.27%
Personalized	9.25%	11.69%
Non-personalized	19.17%	15.16%

**Table 5:** Clarke error grid analysis.

	Zone A	Zone B	Zone C	Zone D	Zone E
Grade A personalized	91.01%	8.19%	0.58%	0.22%	0%
Grade A non-personalized	44.33%	52.66%	2.33%	6.66%	0%
Grade A personalized (after model enhancement)	82.70%	17.30%	0%	0%	0%
Grade A non-personalized (after model enhancement)	75.40%	24.60%	0%	0%	0%

## Data Availability

This study used two datasets from the PhysioNet repository. The Glucose Management in MIMIC-IV dataset (https://physionet.org/content/glucose-management-mimic/1.0.1/) (accessed on 29 October 2025). is available under credentialed access and requires the completion of training and a data use agreement. The MIMIC-III Waveform Database Matched Subset (https://physionet.org/content/mimic3wdb-matched/1.0/) (accessed on 29 October 2025). is openly available.
